# Network Meta‐Analysis: Treatments for Acute Alcohol‐Associated Hepatitis

**DOI:** 10.1111/liv.70922

**Published:** 2026-10-11

**Authors:** Charles Gallaher, Guruprasad P. Aithal, Ashwin Dhanda, Ewan Forrest, James O'Beirne, Ken Liu, Mark Thursz, Richard Parker

**Affiliations:** ^1^ Department of Gastroenterology University Hospital Lewisham London UK; ^2^ Department of Hepatology Nottingham University Hospitals NHS Trust Nottingham UK; ^3^ School of Medicine University of Nottingham Nottingham UK; ^4^ Department of Hepatology University Hospitals Plymouth NHS Trust Plymouth UK; ^5^ Faculty of Health University of Plymouth Plymouth UK; ^6^ Department of Gastroenterology NHS Greater Glasgow and Clyde Glasgow UK; ^7^ School of Medicine University of Glasgow Glasgow UK; ^8^ University of the Sunshine Coast Brisbane Australia; ^9^ Department of Gastroenterology and Hepatology Royal Prince Alfred Hospital Sydney New South Wales Australia; ^10^ Department of Medicine University of Sydney Sydney New South Wales Australia; ^11^ NIHR Imperial Biomedical Research Centre London UK; ^12^ Leeds Liver Unit St James's University Hospital Leeds UK; ^13^ Leeds Institute for Medical Research University of Leeds Leeds UK

**Keywords:** alcohol‐related hepatitis, network meta‐analysis, treatment

## Abstract

**Background:**

Alcohol‐related hepatitis (AH) is an acute form of liver disease with a high short‐term mortality. A variety of treatments have been explored in randomised studies. Recent consensus on study outcomes has identified 90‐day survival as the primary outcome metric.

**Aims:**

This systematic review and network meta‐analysis is to update understanding of the comparative efficacy of treatments for AH, based on 90‐day survival, to inform future Phase III studies.

**Methods:**

PubMed, Ovid‐Medline and Embase were searched for randomised studies of treatments of AH. The risk of bias in included papers was measured using the Cochrane risk of bias 2 tool. Data were synthesised with network meta‐analysis to estimate risk ratio (RR) of survival at 90 days after randomisation. Heterogeneity was assessed with the magnitude of the between‐study variance (tau^2^).

**Results:**

Thirty five studies including 5045 participants were identified that examined 24 treatments for AH. Twenty‐three studies were considered to be at low risk of bias. In network meta‐analysis considering placebo as the reference treatment, only granulocyte colony stimulating factor (GCSF) showed a statistically significant benefit on 90‐day mortality (RR 0.49, 95% CI 0.28–0.84; *p* = 0.009). In subgroup analysis of studies at low risk of bias, no treatments showed a significant effect on mortality at 90 days.

**Conclusions:**

Individual studies are small and heterogeneity is marked; however, some agents show promise to improve survival at 90 days in people with AH. This information should inform adequately powered studies to explore potential treatments for AH.

## Key Points

- Many therapeutic agents have been investigated for the treatment of acute alcohol‐related hepatitis.
- In network meta‐analysis, only GCSF showed an improvement in 90‐day mortality and reduced the incidence of infection.
- Most trials of GCSF have been small and at risk of bias.
- Further analyses that limited network meta‐analysis to trials at low risk of bias did not show a statistically significant benefit of any agents.
- Larger, well‐executed studies are required to develop new treatments for alcohol‐related hepatitis.

## Lay Summary

Alcohol‐related hepatitis (AH) is a sudden and severe type of liver disease caused by alcohol. AH is dangerous with a high risk of death. Many potential treatments for AH have been studied; this work uses a technique called network meta‐analysis to compare all of these treatments against each other. We found that most studies of treatment for AH are small and often at high risk of bias. With this caveat, the results of our study suggest that treatments based on a medicine called granulocyte colony stimulating factor may be effective, but more high‐quality trials are required to confirm this.

## 1 Introduction

Alcohol‐related hepatitis (AH) is an acute form of alcohol‐related liver disease (ALD) characterised by jaundice and liver failure. It has a high short‐term fatality rate which has not significantly changed over recent decades [1]. There is a paucity of effective treatments, with considerable variations both in the quality of evidence for treatments as well as clinical practice between and within institutions.

Both European and US clinical practice guidelines for the management of ALD recommend consideration of corticosteroids (CS), in the absence of contraindications, in patients with severe AH to reduce short‐term mortality [2, 3]. Both guidelines also make a weaker recommendation that the addition of *N*‐acetylcysteine (NAC) to CS therapy may be considered, again with the intention of reducing short‐term mortality, as was demonstrated by Nguyen‐Khac et al. [4]. Guideline‐recommended treatment is otherwise largely supportive, with the pillars being abstinence from alcohol, active nutritional support (targeting daily calorie intake of 35–40 kcal/kg and protein intake of 1.2–1.5 g/kg of body weight as per European guidelines) and identification and treatment of infection, with consideration of liver transplantation in highly selected cases.

Network meta‐analysis allows comparison between treatments that may not have been included in direct head‐to‐head trials. A network meta‐analysis of medical treatments for AH was reported in 2015, when Singh and coworkers did so for drug treatments of severe AH. This was shortly after publication of the landmark STOPAH trial which compared CS, pentoxifylline (PTX) and placebo in different combinations, finding no benefit of PTX and a small benefit of CS on 28‐day mortality [5, 6]. In the decade since, a variety of medical treatments have been explored with some agents showing promise in small‐ or medium‐sized studies. There is new consensus guidance on primary outcome measures in AH studies, moving away from 28‐day survival to 90‐day survival as a standard outcome measure [7]. Islam and colleagues published a systematic review of medical treatments for AH in 2026 [8]. This was primarily a direct meta‐analysis, with a network meta‐analysis reported as a secondary outcome and presented in the supplementary data to the main manuscript. The primary outcome reported was 28‐day, not 90‐day, survival, and the latter has been identified as the optimal primary outcome in a recent international consensus statement [7]. This systematic review and network meta‐analysis updates the understanding of the comparative efficacy of treatments including those that have been reported in the last decade, using the 90‐day survival metric as a primary outcome measure.

## 2 Methods

### 2.1 Search Strategy and Selection Criteria

In this systematic review and network meta‐analysis, we included randomised studies that examined the efficacy of medical (i.e., non‐transplant) treatments for AH in adults. We excluded non‐randomised, retrospective and observational studies. Only studies that were reported in full papers in English were considered. We included all studies from inception of the databases that we searched until the dates of searching.

PubMed and Ovid‐Medline were searched using the search term ((alcoholic hepatitis) OR (alcohol‐associated hepatitis)) OR (alcohol‐related hepatitis), limited to randomised, human studies reported in English. Searches were done on the 12th of February 2025. We also searched citation lists and citing literature of all included studies.

Data were extracted regarding year of publication, average age, gender summary estimates for 30‐, 90‐ and 180‐day survival (from the start of treatment), incidence of infection during the treatment period, baseline biochemical measures and measures of randomisation and blinding. We interrogated supplementary data and derived numerical data from figures where required. We searched trial registers (Clinicaltrials.gov and EU version for unpublished data) and contacted investigators for further information if necessary. Searches were done by one author (RP). Two authors (RP, CG) extracted data. Where differences were found, these authors reviewed these points and differences were resolved through consensus. The study protocol was uploaded to the PROSPERO database of systematic reviews (reference CRD 420251010768).

### 2.2 Statistical Analyses

The primary outcome considered in frequentist network meta‐analysis was 90‐day mortality. Secondary outcomes considered were 1‐month survival (including reported 28‐day and 30‐day mortality) and the incidence of infection. Pre‐planned sub‐group analyses were undertaken that only included studies reported since 2000, and studies considered to be at low risk of bias. We also performed a sub‐group analysis that only included studies with patients with severe disease, defined as a Model for End‐Stage Liver Disease (MELD) score greater than 25. Network meta‐analysis was performed for all included studies. The reference treatment for most analyses was placebo; sub‐group analysis was undertaken using corticosteroid therapy as the reference treatment as this is recommended in specialist guidance.

The effect of each intervention was summarised with a pooled relative risk (RR) with 95% confidence interval (CI), using random effects modelling in meta‐analysis. Forest plots of RR were generated to illustrate the efficacy of each intervention, and network plots were used to illustrate the relationship between tested interventions. All interventions were ranked according to the P‐score. The P‐score is between 0 and 1, based on point estimates and standard errors of the network estimates. P‐scores measure the certainty that one intervention is better than another taking into account all competing interventions, where higher scores suggest that an intervention is likely to be superior.

The risk of bias in included studies was assessed using the RoB 2 tool from the Cochrane group [9]. Two authors (CG, RP) independently assessed studies for risk of bias using this tool to categorise studies as high or low risk of bias. In the case of disagreement between authors, studies were re‐reviewed to reach consensus. Heterogeneity between included studies was measured with tau^2^ to measure the magnitude of between‐study effects. Tau^2^ values above 0.04, 0.16 and 0.36, respectively were considered to represent low, moderate and high levels of heterogeneity. Publication bias was illustrated with funnel plots of effect size versus precision, where symmetry around the effect estimate indicates a low risk of publication bias. Publication bias was also tested with the Egger test, where values < 0.10 are considered significant and indicate possible publication bias. Direct and indirect treatment estimates were assessed by node splitting to indicate local inconsistency in the network meta‐analysis.

All analyses were done in R using the netMeta package, and visualised using ggplot2 packages for data presentation and visualisation [10, 11]. The results are reported in accordance with guidance from the Cochrane handbook and according to PRISMA reporting guidelines [12, 13].

### 2.3 Role of the Funding Source

There was no specific funding source for this study. GPA is supported by the NIHR Nottingham Biomedical Research Centres, and RP is supported by an NIHR SCPRA fellowship (NIHR 304547).

## 3 Results

Literature searches identified a total of 11 094 titles that were reviewed. After review, 11 028 were rejected (1882 duplicates, 9146 not relevant) leaving 64 full manuscripts to be reviewed. Nineteen were considered irrelevant. Further searches of cited and citing literature identified a further four studies not initially found. Sixteen studies did not present 90‐day outcome data and this could not be extracted from Kaplan–Meier plots or provided by the authors. In total 35 studies were included (Figure 1), reporting results from 5045 participants and 24 different treatments for AH. Thirteen studies were placebo‐controlled; others used active comparators, most commonly corticosteroids (Table 1).

**FIGURE 1:**
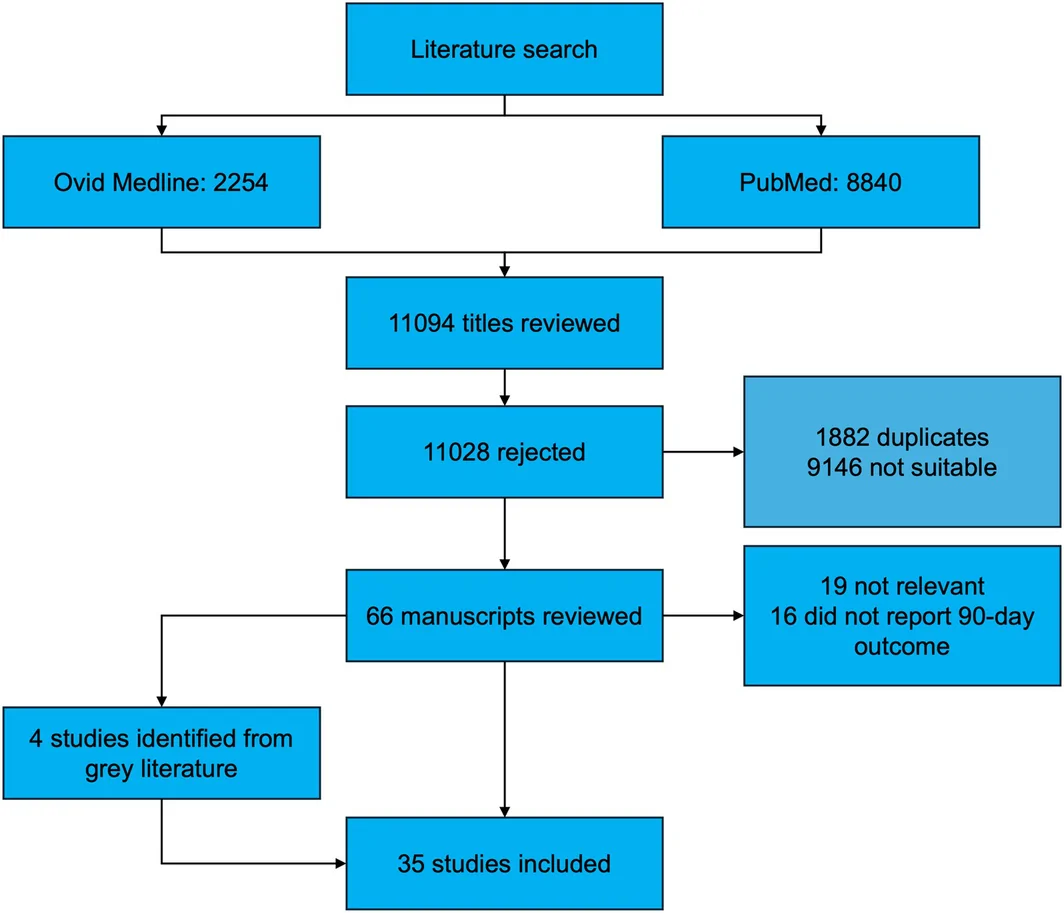
Study flow chart.

**TABLE 1:** Characteristics of included studies.

Trial	*n*	Age (years)	Sex (% male)	MELD	Primary outcome(s)	Treatment arm 1	Survival arm 1 (%)	Treatment arm 2	Survival arm 2 (%)	Treatment arm 3	Survival arm 3	Treatment arm 4	Survival arm 4 (%)	90‐day survival (%)	Incidence of infection (%)
Akriviadis (2000)	101	42	74	28	28‐day survival Progression to hepatorenal syndrome	Pentoxifylline	75.5	Placebo	55.7					65.3	14.3
Boetticher (2008)	48	51	73	25	1‐month survival 6‐month survival	Etanercept	53.8	Placebo	77.2					64.5	34.6
Cabre (2000)	71	48	69	29	28‐day survival	Enteral nutrition	68.5	Corticosteroid	55.5					61.9	42.9
De (2009)	68	47	95	22	3‐month survival	Pentoxifylline	85.2	Corticosteroid	64.7					75.0	
Gawrieh (2024)	147	44	59	24	90‐day survival	IL‐1 inhibition	74.3	Corticosteroid	90.4					82.3	31.1
Higuera‐de la Tijera (2015)	134	44	89		3‐month survival 6‐month survival	Metadoxine + Corticosteroid	31.4	Corticosteroid	20.0	Metadoxine + Pentoxifylline	40.6	Pentoxifylline	68.7	39.5	
Kulkarni (2025)	254	41	98	25	Incidence of infection by day 90	Tapered corticosteroid	86.6	Corticosteroid	83.4					85.0	19.7
Lavekar (2021)	240	48	94	22	1‐month survival 3‐month survival	Pentoxifylline + *N*‐acetylcysteine	80.8	Pentoxifylline	78.3					79.5	
Louvet (2023)	284	53	73	25	60‐day mortality	Corticosteroid + antibiotics	79.5	Corticosteroid	73.9					76.7	29.6
Mathurin (2013)	270	53	60	23	6‐month survival	Corticosteroid + Pentoxifylline	73.6	Corticosteroid	74.4					74.0	
Mendenhall (1984)	263	52	100	25	30‐day survival	Oxandrolone	65.8	Corticosteroid	62.2	Placebo	60.%			62.7	
Mezey (1991)	54	43	59		1‐month survival	Amino acids	64.2	Placebo	65.3					64.8	
Mishra (2024)	126	42	98	25	90‐day survival	GCSF	78.5	Corticosteroid + GCSF	64.2	Corticosteroid	64%			69.0	7.1
Moreno (2010)	56	49	70	22	6‐month survival	*N*‐acetylcysteine	71.4	Enteral nutrition	75.0					73.2	
Moreno (2016)	136	51	64	22	6‐month survival	Enteral nutrition + corticosteroid	60.2	Corticosteroid	61.7					61.0	
Nguyen‐Khac 2011	174	52	52		6‐month survival	Corticosteroid + *N*‐acetylcysteine	77.6	Corticosteroid	66.2					71.8	
Pande (2023)	120	42	97.5	25	90‐day survival	Faecal microbiota transplantation	75.0	Corticosteroid	56.6					65.8	
Park (2014)	121	49	75		1‐month survival	Pentoxifylline	74.1	Corticosteroid	76.2					75.2%	
Phillips (2006)	101	44	59	25	30‐day mortality	Corticosteroid	58.4	*N*‐acetylcysteine	50.0					54.4	45.3
Ramond (1992)	61	48	31	28	2‐month survival	Corticosteroid	84.3	Placebo	44.8					65.5	
Sharma (2017)	50			28	3‐month survival	GCSF	68	Placebo	36.0					52.0	24
Shastry (2019)	28	40		28	28‐day survival	GCSF	64.2	Placebo	28.5					46.4	28
Shiffman (2025)	307	44	57	24	90‐day mortality or liver transplant rate	Larsucosterol 30 mg	85.2	Larsucosterol 90 mg	83.3	Corticosteroid	75.7			81.4	
Sidhu (2012)	70	41	100	26	4‐week survival 6‐month survival	Corticosteroid + Pentoxifylline	36.1	Corticosteroid	26.4					31.4	
Singh (2014)	46	43	100	22	90‐day survival	GCSF + pentoxifylline	78.2	Pentoxifylline	21.7					50.0	
Singh (2018)	57	44	100	25	90‐day survival	GCSF	88.8	GCSF + *N*‐acetylcysteine	68.4	Pentoxifylline	30.0			61.4	
Song (2025)	50	48	70		6‐month transplant‐free survival	Rifaximin	71.4	Corticosteroid	86.2					80.0	
Spahr (2002)	20	53	75		Tolerance to infliximab	Corticosteroid + infliximab	81.8	Corticosteroid	100.0					90.0	18.2
Spahr (2008)	24	54	71		Stem cell mobilisation Liver cell proliferation Liver function	GCSF	76.9	Placebo	63.6					70.8	
Stewart (2007)	70	44	54		6‐month survival	*N*‐acetylcysteine	61.1	Placebo	61.7					61.4	
Szabo (2022)	103	46	61		180‐day survival	IL‐1 inhibition	69.8	Corticosteroid	58.0					64.0	22.6
Tayek (2022)	34	50	100		90‐day survival	Corticosteroid + GCSF	68.7	Corticosteroid	83.3					76.4	18.70
Thompson (2018)	203	46	59		91‐day mortality	Extra‐corporeal liver assist	59.3	Placebo	61.6					60.5	
Thursz (2015)	1099	49	62		28‐day mortality	Corticosteroid	71.1	Pentoxifylline	72.8	Corticosteroid + Pentoxifylline	76.6	Placebo	91.9	78.0	
Vergis (2024)	55	48	54		Histological improvement in inflammation	IL‐1 inhibition	92.8	Placebo	92.5					92.7	3.6

The characteristics of included studies are shown in Table 1. Thirty‐three of the 35 studies reported average age, and the median of the averages was 46.9 (IQR 43.4–49.9) years; 32 studies reported percentage male: the median percentage male was 70.1% (IQR 59.1%–98.8%); thirty‐two studies reported average bilirubin, and the median of the averages was 18.7 mg/dL (IQR 15.5–20.0 mg/dL); 29 studies reported MELD, and the median of the averages was 25.1 (IQR 23.4–26.4). Most studies were small: the median number of patients per study arm was 36 (IQR 21–66), only eight studies had an average of more than 100 patients per arm [6, 14, 15, 16, 17, 18, 19, 20] and one large study accounted for nearly 25% of participants [6]. The risk of bias was considered low in 18 studies, some concerns were noted for 16 studies and two studies were considered to be at high risk of bias (Table 2).

**TABLE 2:**
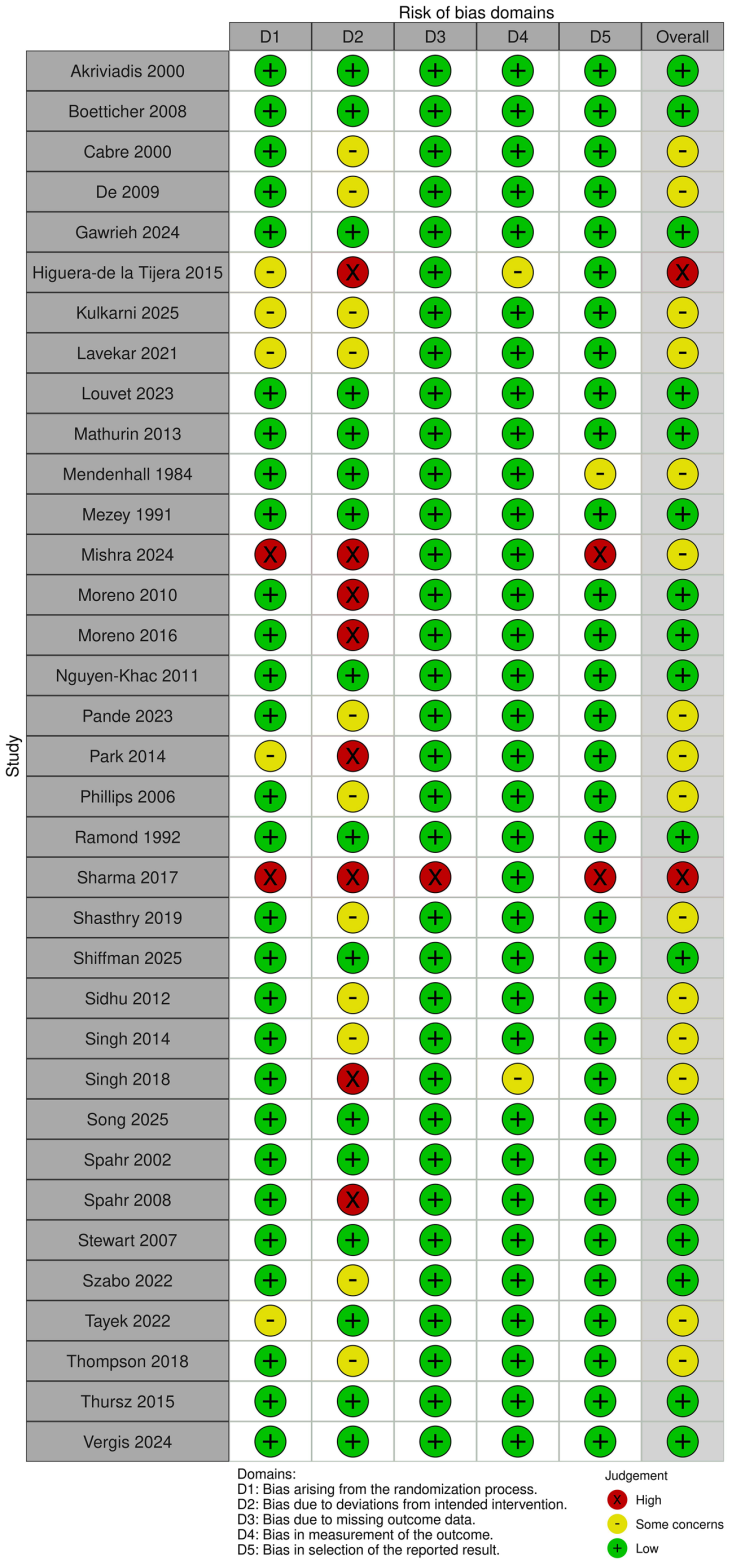
Risk of bias in included studies, assessed with the RoB2 tool.

### 3.1 Survival at 90 Days

Thirty‐five studies were included in this analysis [4, 6, 14, 15, 16, 17, 19, 20, 21, 22, 23, 24, 25, 26, 27, 28, 29, 30, 31, 32, 33, 34, 35, 36, 37, 38, 39, 40, 41, 42, 43, 44, 45, 46, 47], comparing 24 treatments. The overall survival at 90 days was 71% (range 29% to 76%). The network plot for studies contributing to this analysis is shown in Figure 2. Heterogeneity measured with between‐study variance tau^2^ was 0.25, indicating moderate heterogeneity. Publication bias was assessed with a funnel plot showing low asymmetry and a similarly reassuringly non‐significant Egger test (*p* = 0.41) (Supplementary Figure 1). In comparison to placebo, GCSF, based on data from four studies that included 186 participants [28, 35, 36, 42] was the only treatment that showed a statistically significant improved 90‐day survival: RR 0.49, 95% CI 0.26–0.90, *p* = 0.023, P‐score = 0.85. In these studies, absolute risk reduction was 0.24 and number needed to treat was 4.10. Other treatments based on GCSF also suggested benefit but this was not statistically significant: GCSF + pentoxifylline, based on one study that included 41 participants [38] (RR 0.25, 95% CI 0.06–1.03, *p* = 0.056, P‐score 0.931) and GCSF + *N*‐acetylcysteine, based on one study that included 57 patients [39] (RR 0.53, 95% CI 0.15–1.90, *p* = 0.337, P‐score 0.758). Corticosteroids, tested in 29 studies including 2358 participants, were not associated with a significantly lower risk of mortality at 90 days, consistent with evidence from randomised controlled trials and individual patient data meta‐analyses [48] (RR 1.12, 0.69–1.81, *p* = 0.640 P‐score = 0.403). The forest plot for this analysis is shown in Figure 3. All P‐scores for this analysis are shown in Supplementary Table 1. Consistency of the network meta‐analysis was tested by node splitting to compare direct and indirect evidence: the only statistically significant difference was observed for direct and indirect comparisons of corticosteroid + pentoxifylline vs. placebo (direct comparison RR 2.89, indirect comparison RR 0.60, *p* = 0.0225, differences in direct and indirect estimates are shown in Supplementary Figure 2). Whilst estimates for other treatments varied between direct and indirect comparisons this effect did not reach statistical significance suggesting that the network retained consistency.

**FIGURE 2:**
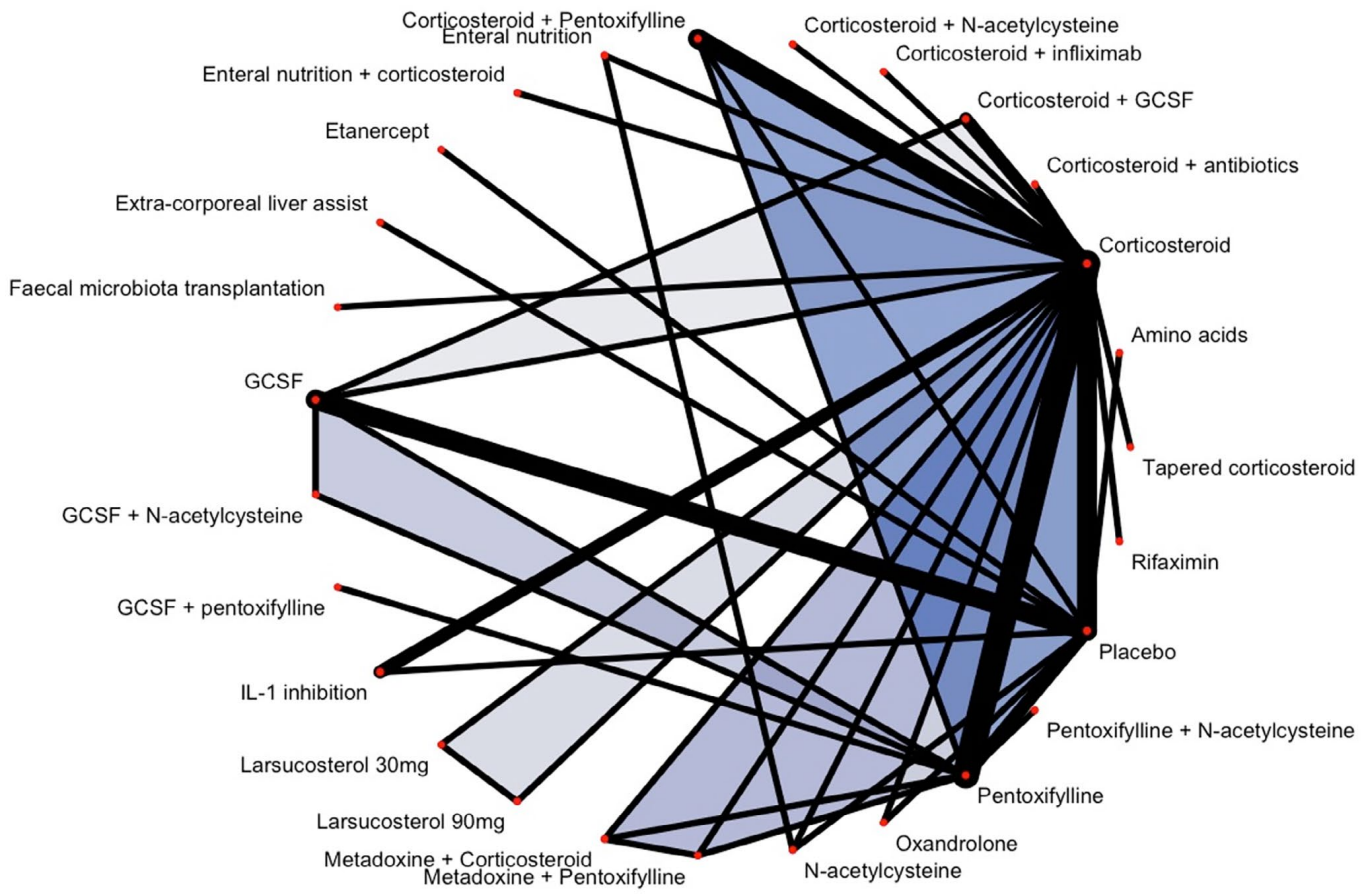
Network plot for studies included in main analysis.

**FIGURE 3:**
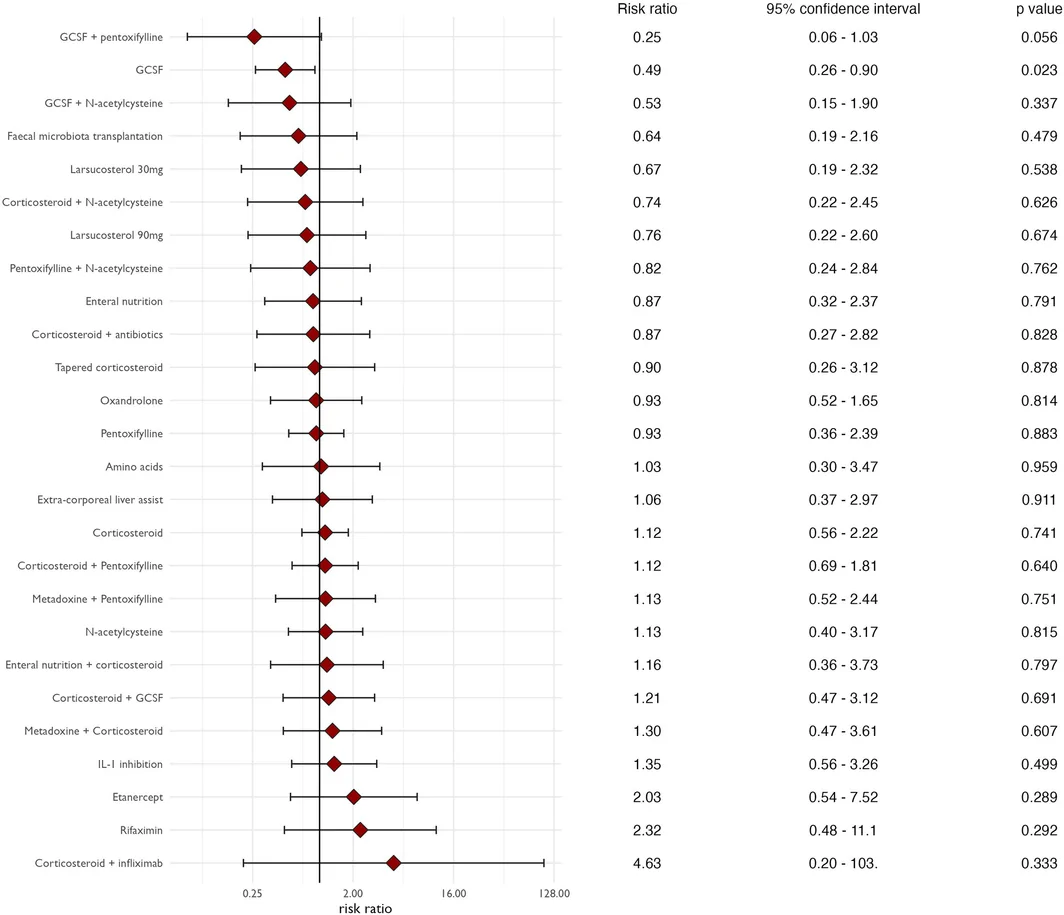
Forest plot of the results of network meta‐analysis of the effect of treatments for alcohol‐related hepatitis on 90‐day mortality.

When only studies at low risk of bias were included, 18 studies [4, 6, 16, 17, 20, 21, 22, 25, 27, 29, 30, 34, 35, 36, 40, 41, 42, 43, 44, 46] comparing 16 treatments were included. The network plot for this analysis is shown in Supplementary Figure 3. Heterogeneity measured with between‐study variance tau^2^ was 0.67 indicating high heterogeneity. Publication bias was assessed with a funnel plot showing low asymmetry and a non‐significant Egger test (*p* = 0.202) (Supplementary Figure 4). In network meta‐analysis, GCSF lost its dominant position and no treatments showed statistically significant evidence of benefit. In comparison to placebo, Larsucosterol 30 mg, in a single study including 307 participants [20] showed the greatest effect on mortality at 90 days (RR 0.56, 0.07–4.22, *p* = 0.580, P‐score = 0.68) (Figure 4A). All P‐scores for this analysis are shown in Supplementary Table 2. To estimate effects in contemporary trials, studies from before 2000 were not included in meta‐analysis, leaving 32 studies [4, 6, 14, 15, 16, 17, 19, 20, 21, 22, 23, 24, 25, 28, 29, 30, 31, 32, 33, 35, 36, 37, 38, 39, 40, 41, 42, 43, 44, 45, 46, 47] comparing 21 treatments. The network plot for this analysis is shown in Supplementary Figure 5. Heterogeneity measured with between‐study variance tau^2^ was 0.21 indicating moderate heterogeneity. Publication bias was assessed with a funnel plot showing asymmetry, albeit with a non‐significant Egger test (*p* = 0.69) (Supplementary Figure 6). In network meta‐analysis, a similar pattern of results was observed to the main analysis where GCSF‐based treatments showed greatest effect. The effect of GCSF on 90 day mortality was of borderline statistical significance: (RR 0.55, 0.30–1.00, *p* = 0.052, P‐score 0.89). All P‐scores from this analysis are shown in Supplementary Table 3. In a supplementary Bayesian network meta‐analysis to support the frequentist primary analysis, GCSF based treatments again tended to show greatest evidence of effect (Supplementary Figure 7) although Metadoxine + Corticosteroid and Metadoxine + Pentoxifylline were also indicated as being treatments more likely to have an effect.

**FIGURE 4:**
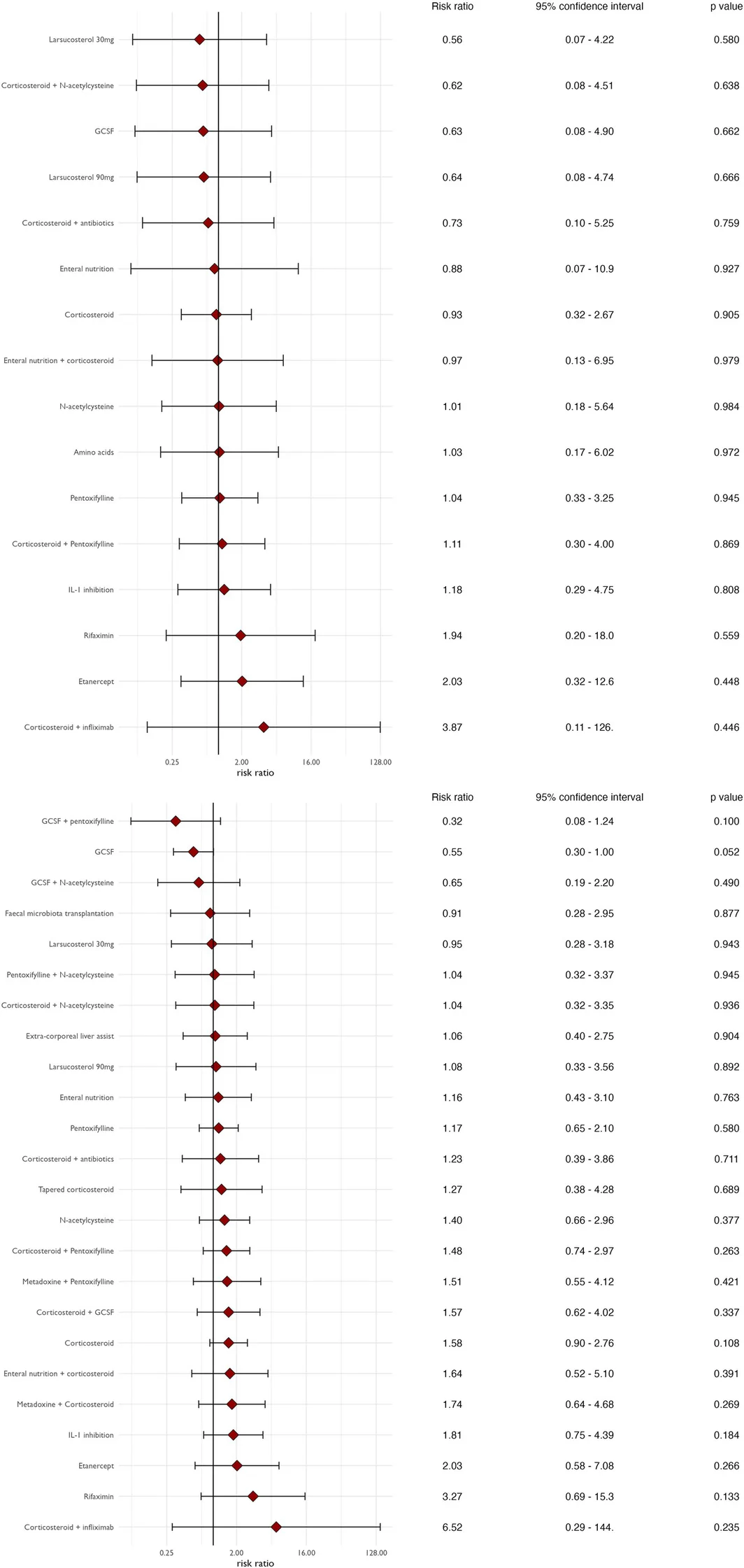
Forest plot of the results of network meta‐analysis of the effect of treatments for alcohol‐related hepatitis on 90‐day mortality, in studies published since 2000.

### 3.2 Secondary Outcomes

#### 3.2.1 One‐Month Survival

The overall survival at one month after admission (including reported 28‐day mortality and 30‐day mortality) was 81%. In network meta‐analysis including 31 studies [4, 6, 14, 15, 16, 17, 19, 21, 22, 23, 24, 25, 27, 28, 29, 30, 31, 32, 33, 34, 35, 36, 37, 38, 41, 43, 44, 45, 46, 47] with relevant data, 21 treatments were compared. The network plot for studies contributing to this analysis is shown in Supplementary Figure 8. Heterogeneity measured with between‐study variance tau^2^ was 0.09, indicating low heterogeneity. Publication bias was assessed with a funnel plot showing low asymmetry and non‐significant Egger test (*p* = 0.20) (Supplementary Figure 9). In comparison to placebo, Corticosteroid and *N*‐acetylcysteine showed the greatest effect on 1‐month mortality (RR 0.22, 0.07–0.68, *p* = 0.008, P‐score 0.93). GCSF + pentoxifylline also showed a significant effect (RR 0.29, 0.08–0.93, *p*—0.038, P‐score 0.87) (Supplementary Figure 10). All P‐scores for this analysis are shown in Supplementary Table 4.

#### 3.2.2 Incidence of Infection

Information regarding the incidence of infection during treatment was available for 15 studies [6, 14, 16, 21, 22, 23, 25, 28, 33, 35, 36, 41, 44, 45, 46] that investigated 10 treatments. The median incidence of infection was 27.4%. The network plot for studies contributing to this analysis is shown in Supplementary Figure 11. Heterogeneity measured with between‐study variance tau^2^ was 0.15, indicating low heterogeneity. Publication bias was assessed with a funnel plot showing low asymmetry; however, Egger test was significant (*p* < 0.001), which raises concern regarding possible publication bias (or small study effects) (Supplementary Figure 12). In network meta‐analysis using placebo as a reference treatment, GCSF, based on data from five studies that included 236 participants [28, 35, 36, 42, 49] significantly reduced the incidence of infection: (RR 0.44, 0.22–0.88, *p* = 0.0021, P‐score = 0.916). No other treatments showed a significant effect to reduce or increase the incidence of infection (Supplementary Figure 13). All P‐scores for this analysis are shown in Supplementary Table 5.

#### 3.2.3 Severe Disease

We repeated the initial network meta‐analysis but only considered studies where the median MELD score was above 25. In this network meta‐analysis, 18 studies were included [14, 16, 19, 21, 22, 23, 26, 28, 31, 33, 34, 35, 36, 37, 39, 40, 44, 45] that considered 14 treatments for AH. The network plot for studies contributing to this analysis is shown in Supplementary Figure 14. Heterogeneity measured with between study variance tau^2^ was 0.21, indicating low heterogeneity. Publication bias was assessed with a funnel plot showing low asymmetry, however, Egger test was significant (*p* < 0.001), raising concerns of possible publication bias (or small study effects) (Supplementary Figure 15). In network meta‐analysis, compared to placebo. GCSF retained a statistically significant association with improved survival (RR 0.38, 0.20–0.74, *p* = 0.004, P‐score 0.77). The forest plot for this analysis is shown in Supplementary Figure 16. All P‐scores are shown in Supplementary Table 6. In an alternative method to address the influence of disease severity on 90 day mortality, network meta‐regression was done using MELD score as a coefficient. This analysis included all studies included in the main analysis, although only a minority of treatments where MELD was consistently reported could be included in meta‐regression: GCSF, enteral nutrition, Pentoxifylline, Corticosteroids, Corticosteroids + pentoxifylline and IL—1 inhibition. A significant effect of MELD was observed on estimates of treatment effect, where the significant positive effect of GCSF was lost (adjusted RR 1.03, 0.98–1.09, *p* = 0.180) and pentoxifylline showed borderline positive effect (RR 0.86, 0.76–0.98, *p* = 0.034) (Figure 5).

**FIGURE 5:**
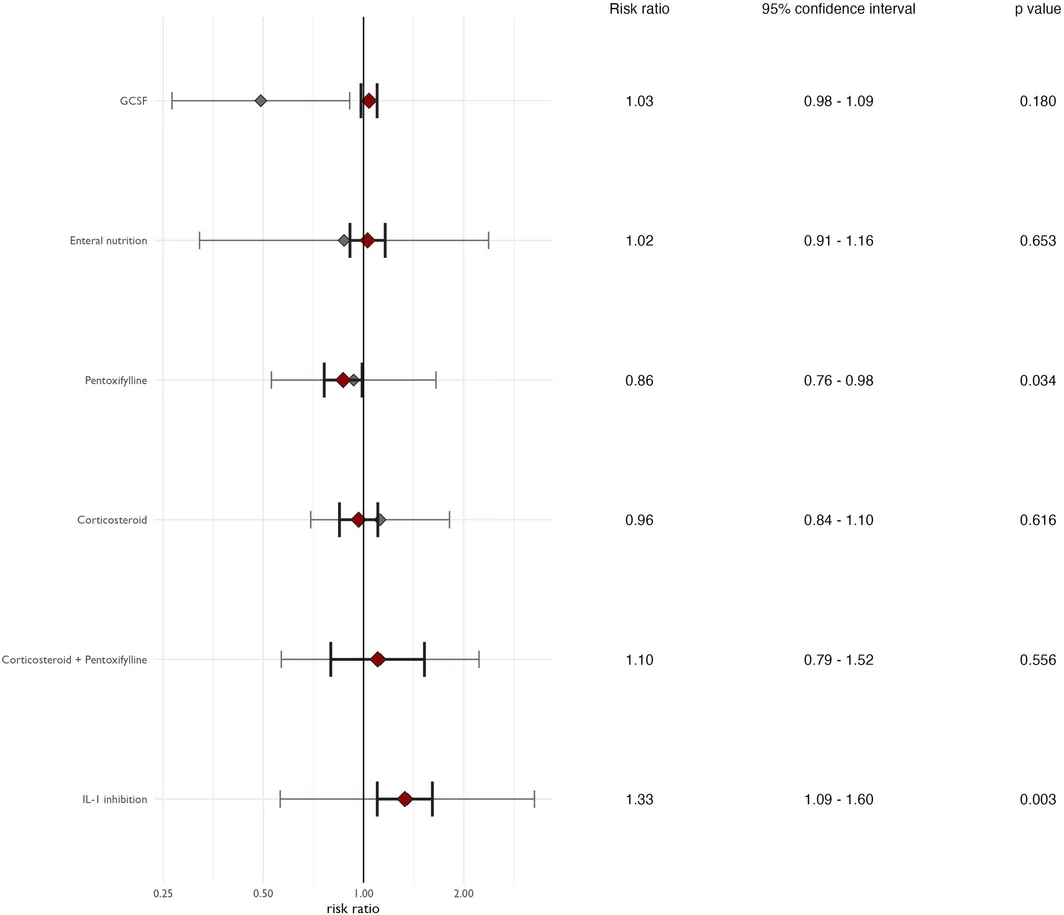
Forest plot of the results of network meta‐analysis of the effect of treatments for alcohol‐related hepatitis on ninety‐day mortality, in studies at low risk of bias.

#### 3.2.4 Corticosteroid as Standard of Care

We repeated the initial network meta‐analysis but only considered studies where corticosteroids were the standard of care, excluding studies with an inactive control arm and using corticosteroid therapy as the reference. In this network meta‐analysis, 24 studies were included [4, 6, 14, 15, 16, 17, 20, 23, 24, 25, 26, 28, 29, 30, 31, 32, 33, 37, 38, 39, 40, 41, 45] that investigated 26 treatments for AH. Heterogeneity measured with between‐study variance tau^2^ was 0.22, indicating low heterogeneity. Publication bias was assessed with a funnel plot showing significant asymmetry, albeit with a reassuringly negative Egger test (*p* = 0.89) (Supplementary Figure 17). In network meta‐analysis using corticosteroid as a reference treatment, GCSF and pentoxifylline, studied in one study including 70 participants [37] showed the greatest effect of mortality at 90 days (RR 0.23, 0.06–0.87, *p* = 0.030, P‐score 0.94). GCSF alone, in one study including 37 participants [39], also showed a significant effect on mortality at 90 days (RR 0.42 0.23–0.76, *p* = 0.004, P‐score = 0.87) (Supplementary Figure 18). All P‐scores from this analysis are shown in Supplementary Table 7.

## 4 Discussion

This systematic review and meta‐analysis of survival in acute alcohol‐associated hepatitis suggests a significant effect of treatments based on GCSF to improve survival and reduce the incidence of infection. The benefit of GCSF used with *N*‐acetylcysteine, pentoxifylline and as monotherapy was consistent in primary and secondary analyses. However, these analyses were based on six small studies (a total of 269 participants, representing only 5% of the overall total number of participants) with only one study at low risk of bias. Once studies with a perceived risk of bias were excluded, GCSF‐based treatments lost their dominant position. In addition, when controlling for disease severity, the estimated benefit of GCSF was seen to be null. Therefore, there is a need for large, well‐conducted studies to provide robust measurement of the effect of GCSF in alcohol‐related hepatitis.

Individual trials and large meta‐analyses have reported survival at 28 days after the initiation of treatment for many years in AH [48]. Recently, an international group of experts recommended that 90 days be considered the primary outcome in studies in AH indicating a need to re‐evaluate current evidence [7]. In this more demanding outcome, corticosteroid treatment, although widely used, does not show a benefit, consistent with large randomised controlled trials [6]. Several agents that demonstrate a survival benefit have often been evaluated in very small trials and cannot be taken as strong evidence of effect. This meta‐analysis does, however, provide a basis for further trial evaluation.

The most obvious limitation to our study is the quality of the underlying evidence. This is illustrated by the fall in the number of included studies when only those at low risk of bias are included: from 35 to 17 studies. When only low‐risk studies are included, many fewer effective agents are identified in network meta‐analysis. Most treatments in the network are supported by only a single, small, randomised trial, which limits the reliability of the estimated treatment effects.

The shift to 90‐day survival as a standard outcome also means that a substantial proportion of the literature became irrelevant as survival at 90 days was not reported. When we considered 28‐day survival in network meta‐analysis using the included studies, a similar pattern of results was seen. Whilst this might suggest that an effect in the short‐term survival may endure to ninety days, analysis of the use of corticosteroids shows that this is not necessarily the case.

The primacy of the 90‐day survival metric is a recent development and whilst many studies report this outcome, only around one third of the included studies reported 90‐day/3‐month mortality as a primary outcome. In studies where 90‐day/3‐month survival was not a pre‐specified outcome, its definition and ascertainment (timing of assessment, completeness of follow‐up, handling of dropouts) may vary between trials, with an attendant risk of selective outcome reporting, increased heterogeneity and a negative impact on the validity of the pooled data. That being said, mortality is a very objective measure, and we do not believe that heterogeneity in definition or reporting is likely to have introduced significant bias. With respect to competing risks, only two of the trials explicitly accounted for liver transplantation within their primary outcomes [20, 31]. Whilst the heterogeneity of the included studies may have been increased by this factor, the number of patients transplanted was low and the probability of this factor causing impactful distortion of the data is low. The possibility of publication bias (or small‐size effects) was raised by four of the funnel plots/Egger analyses, however, on no occasion did both these measures raise concerns on the same analysis, somewhat reducing the degree of concern regarding such bias. The subgroup analysis of studies with patients with severe disease (defined as MELD > 25) is limited by the fact that it was derived from stated and calculated average MELD values at study, rather than patient, level, introducing a risk of ecological bias. Patient‐level data, had it been available, would have allowed severe disease to be more rigorously defined.

Differences in geographical regions and standard‐of‐care practices, which can meaningfully influence outcomes, may have contributed to bias, as may the considerable variability in diagnostic criteria for AH across studies, complicating the assumption that treatment effects are exchangeable. Heterogeneity limits confidence in indirect comparisons in network meta‐analysis because indirect evidence relies on the assumption that the included studies are sufficiently similar.

Our results are, on the surface, discouraging. No agents have strong evidence of benefit of survival at 90 days, and the available literature is often at significant risk of bias. This, in parallel with recent data that confirm that outcomes have not improved over time in AH [1, 50], confirms the urgent need for better evidence and better treatments. More optimistically, our data can provide the basis for future research where the suggestion of benefit in network meta‐analysis can be tested in adequately powered studies. The obvious choice for investigation of existing agents would be treatments that include GCSF, which was frequently dominant in network meta‐analysis. Other new treatments, most obviously Larsucosterol, also showed benefit and current Phase III trials are eagerly awaited. Going forward, evolving methods such as adaptive platform trials and machine‐learning approaches for automated data extraction and predictive modelling may help overcome current evidence gaps. Platform studies are particularly attractive for assessing AH treatments due to their efficiency.

In summary, these data provide a summary of the current available evidence for the treatment of AH against contemporary outcome metrics. Much of the available data are derived from small studies at high risk of bias, although there is evidence of benefit for some treatments. This analysis can support future research into AH (such as by its identification of particularly promising modalities like GCSF and Larsucosterol), a dangerous condition that is frequently fatal and in urgent need of effective treatments and improve confidence in clinical decision‐making.

## Author Contributions

R.P. designed the study, collected data, analysed data and assisted in drafting the manuscript. C.G. collected data, analysed data and assisted in drafting the manuscript. G.P.A., A.D., E.F., J.O.B., K.L., M.T. helped with study design and reviewed the draft manuscript.

## Funding

The authors have nothing to report. G.P.A. is supported by the NIHR Nottingham Biomedical Research Centres. R.P. is supported by an NIHR SCPRA fellowship (NIHR 304547). The views expressed are those of the authors and not necessarily those of the NHS, the NIHR or the Department of Health and Social Care.

## Conflicts of Interest

The authors declare no conflicts of interest.

## Supporting information


**Supplementary Table 1.** P‐scores for each treatment assessed in network meta‐analysis of effect on 90 day mortality.
**Supplementary Table 2**. P‐scores for each treatment assessed in network meta‐analysis of effect on 90 day mortality in studies at low risk of bias.
**Supplementary Table 3**. P‐scores for each treatment assessed in network meta‐analysis of effect on 90 day mortality in studies at published since 2000.
**Supplementary Table 4**. P‐scores for each treatment assessed in network meta‐analysis of effect on 30 day mortality.
**Supplementary Table 5**. P‐scores for each treatment assessed in network meta‐analysis of effect on incidence of infection.
**Supplementary Table 6**. P‐scores for each treatment assessed in network meta‐analysis of effect on 90 day mortality in severe alcohol related hepatitis.
**Supplementary Table 7**. P‐scores from analysis of 90‐day mortality in alcohol related hepatitis, using corticosteroid as the reference treatment.
**Supplementary Figure 1**. Funnel plot for studies included in primary analysis of 90‐day survival.
**Supplementary Figure 2**. Comparison of direct and indirect estimates of effect of treatments for alcohol related hepatitis on 90‐day mortality.
**Supplementary Figure 3**. Network plot of studies considered to be at low risk of bias.
**Supplementary Figure 4**. Funnel plot of studies considered to be at low risk of bias.
**Supplementary Figure 5**. Network plot showing treatment comparisons for studies published since 2000.
**Supplementary Figure 6**. Funnel plot of studies published since 2000.
**Supplementary Figure 7**. Likelihood of effect on 90 day mortality of treatments for alcohol related hepatitis, based on Bayesian network meta‐analysis.
**Supplementary Figure 8**. Network plot showing treatment comparisons for studies that reported 30 day survival.
**Supplementary Figure 9**. Funnel plot for studies reporting 30‐day survival.
**Supplementary Figure 10**. Forest plot showing results of network meta‐analysis of the effect of treatments for AH on 30 day survival.
**Supplementary Figure 11**. Network plot showing treatment comparisons for studies reporting incidence of infection.
**Supplementary Figure 12**. Funnel plot for studies reporting incidence of infection.
**Supplementary Figure 13**. Forest plot showing results of network meta‐analysis of the effect of treatments for AH on the incidence of infection.
**Supplementary Figure 14**. Network plot for studies including patients with more severe disease.
**Supplementary Figure 15**. Funnel plot of studies including patients with severe alcohol related hepatitis.
**Supplementary Figure 16**. Forest plot showing results of network meta‐analysis of the effect of treatments for AH on 90 day survival in studies including patients with severe disease.
**Supplementary Figure 17**. Funnel plot for studies included in network meta‐analysis using corticosteroids as reference treatment.
**Supplementary Figure 18**. Forest plot to show effect of treatments for AH on 90 day survival with corticosteroid as reference treatment.

**Data S1:** PRISMA checklist.

## Data Availability

The data that support the findings of this study are available on request from the corresponding author. The data are not publicly available due to privacy or ethical restrictions.
