# Supplementary material for: Network Meta‐Analysis: Treatments for Acute Alcohol‐Associated Hepatitis
Source: Liver Int. 2026 Oct 11;46(11):e70922. doi: 10.1111/liv.70922 (PMC13655491; doi:10.1111/liv.70922)
Supplement: Supplementary file 2 [file LIV-46-0-s002.docx]

Network meta-analysis: treatments for acute alcohol-associated hepatitis: supplementary material

# Supplementary tables

**Supplementary table 1:** P-scores for each treatment assessed in network meta-analysis of effect on 90 day mortality

**Supplementary table 2:** P-scores for each treatment assessed in network meta-analysis of effect on 90 day mortality in studies at low risk of bias

**Supplementary table 3:** P-scores for each treatment assessed in network meta-analysis of effect on 90 day mortality in studies at published since 2000

**Supplementary table 4:** P-scores for each treatment assessed in network meta-analysis of effect on 30 day mortality

**Supplementary table 5:** P-scores for each treatment assessed in network meta-analysis of effect on incidence of infection

**Supplementary table 6:** P-scores for each treatment assessed in network meta-analysis of effect on 90 day mortality in severe alcohol related hepatitis

**Supplementary table 7**: P-scores from analysis of 90-day mortality in alcohol related hepatitis, using corticosteroid as the reference treatment

# Supplementary figures

**Supplementary figure 1:** funnel plot for studies included in primary analysis of 90-day survival

**Supplementary figure 2**: comparison of direct and indirect estimates of effect of treatments for alcohol related hepatitis on 90-day mortality

**Supplementary figure 3:** network plot of studies considered to be at low risk of bias

**Supplementary figure 4**: funnel plot of studies considered to be at low risk of bias

**Supplementary figure 5**: network plot showing treatment comparisons for studies published since 2000

**Supplementary figure 6**: funnel plot of studies published since 2000

**Supplementary figure 7:** likelihood of effect on 90 day mortality of treatments for alcohol related hepatitis, based on Bayesian network meta-analysis

**Supplementary figure 8:** network plot showing treatment comparisons for studies that reported 30 day survival

**Supplementary figure 9**: funnel plot for studies reporting 30-day survival

**Supplementary figure 10:** forest plot showing results of network meta-analysis of the effect of treatments for AH on 30 day survival

**Supplementary figure 11**: network plot showing treatment comparisons for studies reporting incidence of infection

**Supplementary figure 12**: funnel plot for studies reporting incidence of infection

**Supplementary figure 13:** forest plot showing results of network meta-analysis of the effect of treatments for AH on the incidence of infection

**Supplementary figure 14**: network plot for studies including patients with more severe disease

**Supplementary figure 15**: funnel plot of studies including patients with severe alcohol related hepatitis

**Supplementary figure 16:** forest plot showing results of network meta-analysis of the effect of treatments for AH on 90 day survival in studies including patients with severe disease

**Supplementary figure 17:** funnel plot for studies included in network meta-analysis using corticosteroids as reference treatment

**Supplementary figure 18:** forest plot to show effect of treatments for AH on 90 day survival with corticosteroid as reference treatment
